# Preparation of Cl-Doped g-C_3_N_4_ Photocatalyst and Its Photocatalytic Degradation of Rhodamine B

**DOI:** 10.3390/molecules30091910

**Published:** 2025-04-25

**Authors:** Jing Zhang, Lixia Wang, Yang Li, Yuhong Huang, Renbin Song, Chen Cheng, Qian Luo, Ruiqi Zhai, Yijie Meng, Peixin Zhang, Qiang Ma, Yingjie Zhang

**Affiliations:** 1 College of Agriculture and Biological Science, Dali University, Dali 671003, China; zj2452488891@163.com (J.Z.); lq816615@163.com (Q.L.); zhairuiqi2023@163.com (R.Z.); asd2939197052@163.com (Y.M.); 17869016909@163.com (P.Z.); 2 Kunming Dianchi and Plateau Lakes Institute, Kunming 650228, China; 15687135855@163.com (L.W.); scorpioboygood@126.com (Y.L.); hyh696158@163.com (Y.H.); srbkmstdianchi@163.com (R.S.); 3 School of Architecture and Civil Engineering, Chengdu University, Chengdu 610106, China; maqiang@cdu.edu.cn

**Keywords:** graphitic phase carbon nitride, Rhodamine B, photocatalysis, Cl-doped photocatalyst

## Abstract

The increasing global demand for clean water is driving the development of advanced wastewater treatment technologies. Graphitic carbon nitride (g-C_3_N_4_) has emerged as an efficient photocatalyst for degrading organic pollutants, such as synthetic dyes, due to its exceptional thermo-chemical stability. However, its application is limited by an insufficient specific surface area, low photocatalytic efficiency, and an unclear degradation mechanism. In this study, we aimed to enhance g-C_3_N_4_ by doping it with elemental chlorine, resulting in a series of Cl-C_3_N_4_ photocatalysts with varying doping ratios, prepared via thermal polymerization. The photocatalytic activity of g-C_3_N_4_ was assessed by measuring the degradation rate of RhB. A comprehensive characterization of the Cl-C_3_N_4_ composites was conducted using SEM, XRD, XPS, PL, DRS, BET, EPR, and electrochemical measurements. Our results indicated that the optimized 1:2 Cl-C_3_N_4_ photocatalyst exhibited exceptional performance, achieving 99.93% RhB removal within 80 min of irradiation. TOC mineralization reached 91.73% after 150 min, and 88.12% removal of antibiotics was maintained after four cycles, demonstrating the excellent stability of the 1:2 Cl-C_3_N_4_ photocatalyst. Mechanistic investigations revealed that superoxide radicals (·O_2_^−^) and singlet oxygen (^1^O_2_) were the primary reactive oxygen species responsible for the degradation of RhB in the chlorine-doped g-C_3_N_4_ photocatalytic system.

## 1. Introduction

With the rapid advancement of technological innovation and economic expansion, global ecosystems have experienced unparalleled environmental degradation. Concurrently, industrial pollution has escalated, posing significant risks to both human health and biodiversity conservation. These dual pressures highlight the urgent need for coordinated global efforts to achieve sustainable development [1]. One of the most challenging issues is the management of industrial pollutants such as Rhodamine B (RhB), a basic organic dye derived from trinitrobenzene. Widely used as a coloring agent in the textile, paper, and cosmetics industries, RhB is frequently found in industrial wastewater [2]. Rhodamine-based dyes, particularly RhB, are recognized as persistent environmental pollutants due to their teratogenic and carcinogenic properties [3]. This pressing public health concern has prompted extensive scientific research aimed at developing advanced technologies for the treatment of dye-contaminated wastewater [4,5]. RhB possesses a highly stable chemical structure, and its residues in aquatic environments represent a significant threat to human health [6,7]. Currently, both domestic and international methods for treating dye wastewater primarily include biodegradation [8,9], physical adsorption [10,11], membrane separation [12], and various chemical approaches. However, these methods are associated with several limitations, such as high treatment costs, harsh experimental conditions, secondary pollution, and incomplete degradation [13].

Photocatalytic technology is currently regarded as a promising approach for pollutant treatment [14]. Photocatalytic technology can convert organic pollutants into CO_2_ and water with the assistance of semiconductor photocatalysts, without generating toxic by-products [15,16]. Since the discovery of carbon nitride in 1989, g-C_3_N_4_ has gained widespread use in photocatalysis due to its ease of preparation, non-toxicity, good stability, and excellent response to visible light [17,18], however, the narrow bandgap of g-C_3_N_4_, its limited utilization of visible light, and the high recombination rate of photogenerated charge carriers hinder its practical application [19,20]. As a result, a single g-C_3_N_4_ semiconductor is often insufficient for efficient photocatalytic degradation of organic pollutants. In recent years, research has focused on modifying g-C_3_N_4_ to address these limitations, primarily through morphological and structural modifications, non-metallic doping, metal doping, and composites with other semiconductor materials [21,22,23,24,25,26]. Non-metal doping involves the substitution of C atoms at the C1 and C2 positions or N atoms at the N1, N2, and N3 positions in g-C_3_N_4_ by impurity elements [27,28]. This process effectively tunes the specific surface area, electronic structure, and band gap of g-C_3_N_4_, thereby broadening its visible light absorption range, inhibiting the recombination of photo-induced electrons and holes, and consequently enhancing its photocatalytic performance [29,30]. Compared to metal doping, non-metal doping avoids the risk of secondary metal pollution, making it a more environmentally sustainable option with greater potential for practical applications [31,32,33]. Tong et al. [34] prepared TiO_2_/g-C_3_N_4_ composites via hydrothermal synthesis and demonstrated that TiO_2_/g-C_3_N_4_ achieved near-complete photocatalytic degradation of RhB within 50 min. Yi et al. [35] synthesized S/Cl co-doped g-C_3_N_4_ photocatalysts using thiourea and ammonium chloride as precursors. The S/Cl-g-C_3_N_4_ composite exhibited excellent photocatalytic activity, characterized by favorable redox potentials, enhanced light absorption, and a larger specific surface area. Furthermore, the introduction of chlorine enabled the formation of chlorine-based reactive species during the reaction. In another study, Hu et al. [36] prepared PS co-doped g-C_3_N_4_ using melamine, hexachlorotriphosphonitrile, and sulfur as precursors. The resulting PSCN samples exhibited reduced photoluminescence emission intensity, which indicated the suppression of photogenerated charge recombination and consequently enhanced photocatalytic degradation activity. In this study, g-C_3_N_4_ was modified with non-metallic element Cl by the thermal polymerization method, and the photocatalytic materials were characterized by XRD, SEM, XPS, PL, EPR, etc. RhB was taken as the target pollutant to investigate the effect of modified photocatalytic materials on the degradation performance of RhB. The effect of the modified photocatalytic materials on the degradation of RhB was investigated. By exploring a green and efficient method for the degradation of RhB, an effective way for the management of RhB in the aquatic environment can be provided.

## 2. Results and Discussion

### 2.1. Catalyst Characterization

**Figure 1:**
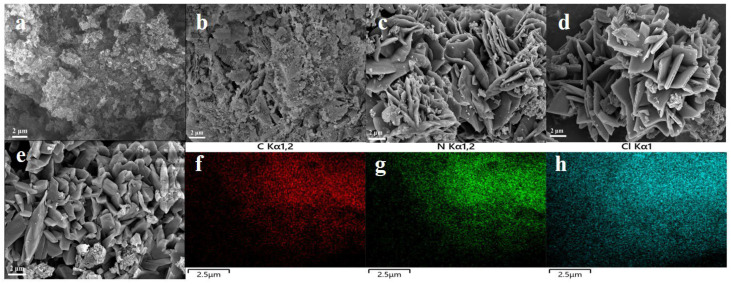
(**a**) SEM image of (**a**) g-C_3_N_4_, (**b**) 1:1 Cl-C_3_N_4_, (**c**) 1:2 Cl-C_3_N_4_, (**d**) 1:5 Cl and (**e**) 2:1 Cl-C_3_N_4_ at 2 μm resolution; and (**f**–**h**) Elemental mapping of 1:2 Cl-C_3_N_4_.

The surface morphology of both the unmodified photocatalytic material (g-C_3_N_4_) and the modified photocatalyst (Cl-C_3_N_4_) was characterized using SEM. As shown in Figure 1a, the unmodified g-C_3_N_4_ exhibits a distinct, irregular multilayer lamellar stacked bulk structure, which can be attributed to the direct thermal polymerization of g-C_3_N_4_ from melamine. In contrast, Figure 1b–e reveal that, compared to the unmodified g-C_3_N_4_, the samples with various doping ratios display more pronounced lamellar structures and increased delamination, featuring thinner sheets, smoother surfaces, and more irregular mesopores. This enhanced lamellar structure is more favorable for photocatalytic reactions, as it facilitates the easier transfer of photogenerated electrons [37]. The presence of ammonium chloride may contribute to the formation of this structure, as it functions both as a dopant for the chlorine element and as a soft template during the preparation process [38]. Additionally, the EDS spectra of the 1:2 Cl-C_3_N_4_ shown in Figure 1f–h reveal the presence of C, N, and Cl elements, further confirming the uniform distribution of the chlorine element within the g-C_3_N_4_ particles.

**Figure 2:**
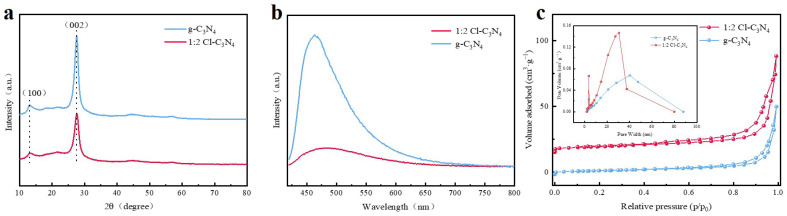
(**a**) XRD patterns of g-C_3_N_4_ and 1:2 Cl−C_3_N_4_; (**b**) PL spectra of g-C_3_N_4_ and 1:2 Cl-C_3_N_4_; (**c**) N_2_ adsorption–desorption isotherms and distribution of pore size plots of g-C_3_N_4_ and 1:2 Cl-C_3_N_4_.

The X-ray diffractograms of g-C_3_N_4_ and 1:2 Cl-C_3_N_4_ are shown in Figure 2a. For g-C_3_N_4_, two distinct peaks are observed, both corresponding to typical diffraction features of the g-C_3_N_4_ structure. The peak at 27.5° is assigned to the characteristic (002) diffraction peak of g-C_3_N_4_, which arises from the interlayer stacking of the conjugated aromatic lamellae. Meanwhile, the peak at 13.1° corresponds to the (100) diffraction peak, associated with the stacking of nitrogen holes in the basic unit of the heptazinium ring structure [39]. In comparison to g-C_3_N_4_, the (002) diffraction peak of 1:2 Cl-C_3_N_4_ is shifted to a lower angle, indicating a slight enlargement of the interlayer spacing in 1:2 Cl-C_3_N_4_. This expansion is attributed to the structural modification, likely resulting from chlorine elemental doping, which leads to the increased interlayer spacing [40].

The separation efficiency of photogenerated electron-hole pairs (e^−^/h^+^) for the samples was investigated using photoluminescence (PL) spectroscopy. This analysis revealed a decrease in fluorescence intensity, accompanied by an enhancement in the photogenerated carrier separation ability of the photocatalysts [41]. The PL intensities of g-C_3_N_4_ and 1:2 Cl-C_3_N_4_ are compared in Figure 2b. The PL spectra of both g-C_3_N_4_ and 1:2Cl-C_3_N_4_ are similar, with a peak at 440 nm, which is characteristic of g-C_3_N_4_ [42]. However, the PL intensity of 1:2 Cl-C_3_N_4_ is significantly lower than that of g-C_3_N_4_, further indicating that chlorine doping enhances the separation efficiency of photogenerated carriers at the same excitation wavelength, effectively reducing the recombination of electron-hole pairs. Figure 2c presents the nitrogen adsorption–desorption isotherms and pore size distribution curves for both g-C_3_N_4_ and 1:2 Cl-C_3_N_4_. Both the unmodified g-C_3_N_4_ and 1:2 Cl-C_3_N_4_ exhibit typical Type IV adsorption isotherms with H3-type hysteresis loops, indicating the presence of mesoporous structures (pore size range: 2.00–50.00 nm) in both samples. The specific surface areas of g-C_3_N_4_ and 1:2 Cl-C_3_N_4_ are 9.95 m^2^/g and 15.17 m^2^/g, respectively, with average pore sizes of 30.13 nm and 28.18 nm, respectively. The enhanced adsorption capacity of 1:2 Cl-C_3_N_4_, as compared to g-C_3_N_4_, suggests that the modification has a noticeable effect on the specific surface area of g-C_3_N_4_. This increase in surface area provides additional active sites for adsorption and surface reactions, thereby significantly enhancing photocatalytic performance.

**Figure 3:**
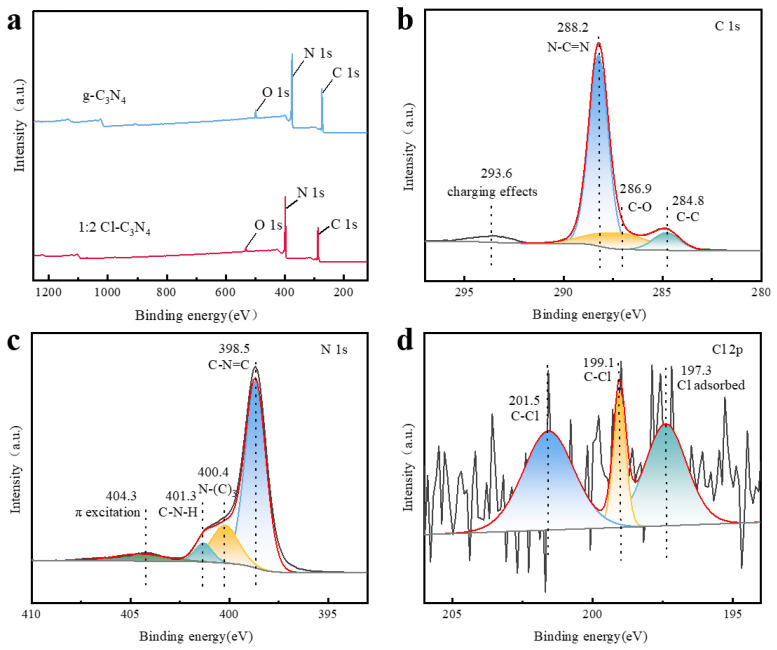
XPS spectra (**a**) XPS full spectra of 1:2 Cl-C_3_N_4_ and g-C_3_N_4_; (**b**) C 1s fine XPS spectrum of 1:2 Cl-C_3_N_4_; (**c**) N 1s fine XPS spectrum of 1:2 Cl-C_3_N_4_; (**d**) Cl 2p fine XPS spectrum of 1:2 Cl-C_3_N_4_.

The catalytic materials were characterized using X-ray photoelectron spectroscopy (XPS), and the full XPS spectra of 1:2 Cl-C_3_N_4_ and g-C_3_N_4_ were obtained. As shown in the full spectrum in Figure 3a, the primary elements—C, N, and O—were detected in both 1:2 Cl-C_3_N_4_ and g-C_3_N_4_. No significant shifts in the binding energies of C 1s, N 1s, and O 1s were observed, suggesting that the chemical states of carbon, nitrogen, and oxygen in the modified 1:2 Cl-C_3_N_4_ are nearly identical to those in g-C_3_N_4_. The chlorine peak in the full spectrum of 1:2 Cl-C_3_N_4_ was not clearly visible, likely due to the thermal decomposition of ammonium chloride at elevated temperatures. Additionally, the higher nitrogen content in melamine may preferentially bind with hydrogen ions (H^+^), thus inhibiting the stable presence of chloride ions (Cl^−^). These two factors likely contribute to a reduction in chloride content, making the chloride peak nearly undetectable in the XPS spectrum, which results in weak elemental peaks in the full spectrum [43,44]. As shown in Figure 3b, the C 1s fine spectrum of 1:2Cl-C_3_N_4_ reveals three distinct carbon peaks. The peak at 284.8 eV is assigned to C-C bonding, while the peaks at 286.9 eV and 288.2 eV are attributed to C-O and N-C=N bonding, respectively. Figure 3c presents the fine spectrum of nitrogen, where four peaks are observed at 398.5 eV, 400.4 eV, 401.3 eV, and 404.3 eV. The strongest peak at 398.5 eV is attributed to sp2-hybridized aromatic nitrogen bound to the carbon atoms in C-N=C [45,46]. The peak at 400.4 eV corresponds to the bridging nitrogen atom (N-(C)_3_), while the peak at 401.3 eV is assigned to C-N-H bonding. A relatively weak peak at 404.3 eV is mainly attributed to π-excitation [47]. The fine spectrum of chlorine, shown in Figure 3d, reveals a small amount of chlorine doping, with characteristic C-Cl peaks at 199.1 eV and 201.5 eV, as well as peaks due to surface-adsorbed chloride ions at 197.3 eV.

**Figure 4:**
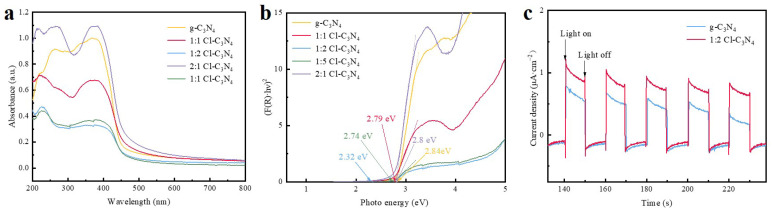
(**a**) The DRS spectra and (**b**) the calculated bandgap energies diagram of g-C_3_N_4_, 1:1 Cl-C_3_N_4_, 1:2 Cl-C_3_N_4_, 1:5 Cl-C_3_N_4_, and 2:1 Cl-C_3_N_4_; (**c**) transient photocurrent profiles of g-C_3_N_4_ and 1:2 Cl-C_3_N_4_.

The optical absorption properties of the synthesized photocatalysts were systematically investigated using solid-state UV-Vis diffuse reflectance spectroscopy (DRS). As shown in Figure 4a, a comparative analysis of the absorption spectra reveals that all chlorine-doped photocatalysts exhibit significantly enhanced visible light absorption within the 380–780 nm range compared to pristine g-C_3_N_4_. Notably, the 1:2 Cl-C_3_N_4_ composite shows a distinct bathochromic shift in its absorption edge, which can be attributed to the successful incorporation of chlorine atoms into the g-C_3_N_4_ framework. This shift suggests that chlorine doping effectively modifies the electronic structure of g-C_3_N_4_, narrowing the bandgap and consequently broadening the photocatalyst’s spectral response range. The observed enhancement in visible light absorption is particularly advantageous for photocatalytic applications, as it promotes better utilization of solar energy and may improve photocatalytic performance under visible light irradiation. The band gaps were further determined using the Kubelka-Munk equation [48], as shown in Figure 4b. The band gap energies for g-C_3_N_4_, 1:1 Cl-C_3_N_4_, 1:2 Cl-C_3_N_4_, 1:5 Cl-C_3_N_4_, and 2:1 Cl-C_3_N_4_ were found to be 2.84 eV, 2.79 eV, 2.32 eV, 2.74 eV, and 2.8 eV, respectively. Notably, the band gap of 1:2 Cl-C_3_N_4_ is reduced by 0.52 eV compared to the pristine g-C_3_N_4_. This reduction further supports the notion of band gap modulation during the composite formation, indicating that the incorporation of chlorine alters the electronic band structure of g-C_3_N_4_. Consequently, the decrease in band gap energy leads to a narrowing of the forbidden band width [49].

To further evaluate the separation and transfer efficiency of charge carriers, transient photocurrent measurements were performed under visible light irradiation. Figure 4c presents a comparison of the transient photocurrent responses of g-C_3_N_4_ and 1:2 Cl-C_3_N_4_. Upon illumination, the photocurrent of both samples rapidly increased to its maximum value and then stabilized. When the light was turned off, the photocurrent promptly decreased to the background level. Notably, 1:2 Cl-C_3_N_4_ exhibited a higher photocurrent than g-C_3_N_4_, indicating enhanced separation and transfer of photogenerated electron-hole pairs in 1:2 Cl-C_3_N_4_. These results clearly demonstrate that the incorporation of chlorine significantly improves the light-harvesting capability, charge transfer, and charge separation efficiency of g-C_3_N_4_. Consequently, superior photocatalytic performance can be anticipated.

### 2.2. Effect of Different Factors on Catalytic Activity

#### 2.2.1. Different Ratios of Catalysts

**Figure 5:**
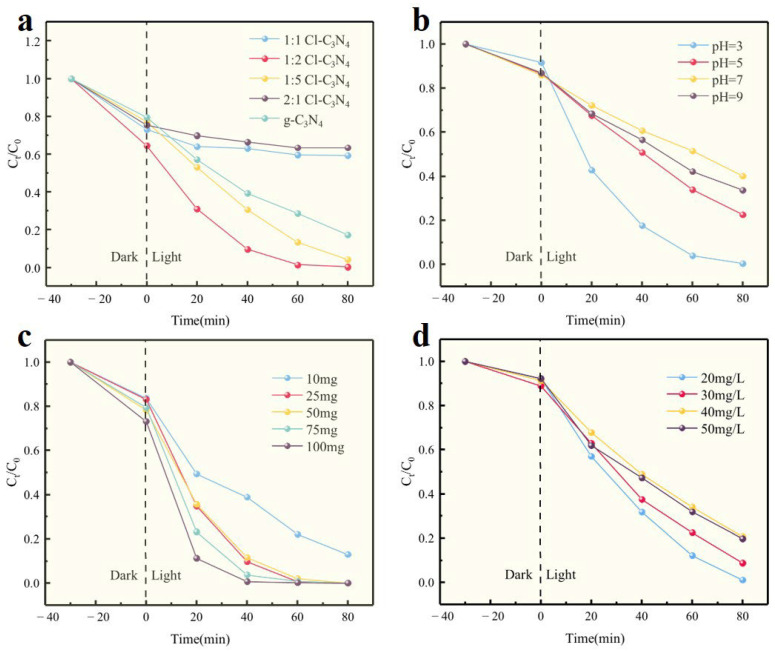
Efficiency of visible light degradation of RhB by 1:2 Cl-C_3_N_4_ under different conditions: (**a**) different catalysts, (**b**) pH, (**c**) dosing, (**d**) initial RhB concentration.

To evaluate the potential application of the investigated catalysts in environmental remediation processes, photocatalytic experiments were conducted using dissolved RhB in water under visible light irradiation. As shown in Figure 5a, the degradation rates of g-C_3_N_4_, 1:1 Cl-C_3_N_4_, 1:2 Cl-C_3_N_4_, 1:5 Cl-C_3_N_4_, and 2:1 Cl-C_3_N_4_ after 80 min were 82.82%, 40.65%, 99.79%, 95.81%, and 36.52%, respectively. The photocatalytic performance was optimal when the mass ratio of ammonium chloride to melamine was 1:2, resulting in a RhB degradation rate of 98.09% after 80 min. In contrast, as the amount of ammonium chloride doping increased, the photocatalytic performance of the samples decreased. Specifically, when the mass ratio of ammonium chloride to melamine was 2:1, the degradation rate of RhB decreased to 36.52% at 80 min. These results clearly demonstrate that an appropriate level of chlorine doping can effectively enhance the photocatalytic performance of graphitic-phase carbon nitride. However, higher chlorine doping ratios appear to have a detrimental effect on photocatalytic degradation, likely due to the fact that optimal chlorine doping reduces the bandgap of the photocatalyst, thereby promoting the generation of photogenerated electrons [50]. Therefore, samples with a 1:2 doping mass of ammonium chloride and melamine were selected as photocatalysts for subsequent studies.

#### 2.2.2. pH

In aqueous systems, pH is a critical factor influencing the reaction, as it affects the dissociation behavior and the types of free radicals involved in the photocatalytic degradation of RhB. To investigate the impact of pH on RhB degradation, the effect of the initial solution pH was examined using 1:2 Cl-C_3_N_4_ as the photocatalyst. The results are presented in Figure 5b. Photocatalytic degradation experiments were conducted at pH values of 3.0, 5.0, 7.0, and 9.0, and it was observed that the initial pH significantly influenced RhB degradation. At pH 3.0, 5.0, 7.0, and 9.0, the degradation rates of RhB after 80 min were 99.6%, 77.46%, 59.96%, and 66.36%, respectively. These results demonstrate that RhB degradation was most effective at pH 3.0, with the degradation rate decreasing as the pH increased. This trend may be attributed to changes in the morphology or charge distribution on the surface of the photocatalyst due to varying pH levels. Therefore, pH 3.0 was selected as the optimal value for subsequent experiments.

#### 2.2.3. Different Dosage

The chlorine-doped graphitic carbon nitride photocatalyst plays a crucial role in the photocatalytic degradation of pollutants, and the catalyst dosage is inherently linked to the degradation rate of pollutants. To investigate the relationship between the dosage of chlorine-doped graphitic carbon nitride photocatalyst and the degradation rate of RhB, the influence of different catalyst dosages (10 mg, 25 mg, 50 mg, 75 mg, and 100 mg) on RhB degradation was examined. The results are shown in Figure 5c. As the dosage of 1:2 Cl-C_3_N_4_ increased, the removal of RhB initially increased and then decreased. The degradation rates were 84.53% at 10 mg, 99.93% at 25 mg, 99.93% at 50 mg, 99.93% at 75 mg, and 99.91% at 100 mg. This trend may be attributed to a high catalyst dosage, which could hinder the transmission of visible light, thereby reducing the photocatalytic activity [51]. Considering the cost and other aspects, the optimum dosage of 25 mg of 1:2 Cl-C_3_N_4_ was selected.

#### 2.2.4. Initial RhB Concentration

The concentration of pollutants is a crucial factor influencing the degradation of pollutants. To investigate the effect of the initial RhB concentration on its degradation rate in a 1:2 Cl-C_3_N_4_ system, we examined the degradation rates at various initial RhB concentrations (20 mg/L, 30 mg/L, 40 mg/L, and 50 mg/L). The results are presented in Figure 5d. As shown, the degradation rates of RhB at initial concentrations of 20 mg/L, 30 mg/L, 40 mg/L, and 50 mg/L were 98.95%, 91.23%, 79.28%, and 80.24%, respectively. Notably, higher RhB concentrations resulted in lower degradation rates. The reduced degradation efficiency at higher concentrations may be attributed to the limited availability of active sites and reactive species within the system, leading to a decreased degradation rate and overall efficiency. Furthermore, the higher concentration of RhB resulted in the generation of more intermediates, which competed with RhB for degradation, thereby contributing to the lower degradation rate at elevated concentrations [52]. Therefore, the experiments were conducted using an initial RhB concentration of 20 mg/L. Based on the results obtained, the optimal degradation conditions were established as follows: a catalyst ratio of 1:2, a catalyst dosage of 25 mg, a pH of 3, and an initial RhB concentration of 20 mg/L.

### 2.3. TOC Testing and Cycling Stability of 1:2 Cl-C_3_N_4_

**Figure 6:**
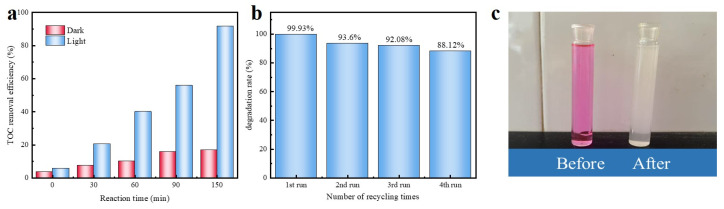
(**a**) TOC removal efficiency (initial concentration: 20 mg/L, dosage: 25, pH = 3.0), (**b**) Four-cycle degradation experiment, (**c**) Comparison of RhB before and after photocatalytic reaction.

In this study, the mineralization ability of the 1:2 Cl-C_3_N_4_ catalyst for RhB was evaluated through total organic carbon (TOC) removal. As shown in Figure 6a, the TOC mineralization was only 16.75% after 150 min of dark reaction under the conditions of 25 mg of catalyst and an initial RhB concentration of 20 mg/L. In contrast, the mineralization efficiency reached 91.73% under the same conditions in the photocatalytic system. This comparative experiment highlights the critical role of light in enhancing the mineralization capacity of 1:2 Cl-C_3_N_4_, with the photocatalytic process improving efficiency by a factor of 5.47 compared to the dark reaction. Under visible light irradiation, the RhB molecules were decomposed into smaller organic molecules, indicating that 1:2 Cl-C_3_N_4_ exhibits excellent mineralization ability. Most of the RhB was mineralized into CO_2_ and H_2_O, further confirming the superior photocatalytic performance of 1:2 Cl-C_3_N_4_ [53].

The stability and reusability of a photocatalyst are crucial factors in determining its potential for practical engineering applications. As shown in Figure 6b, the degradation activity of 1:2 Cl-C_3_N_4_ decreased only slightly, from 99.93% to 88.12%, after four cycles of degradation experiments. This indicates that 1:2 Cl-C_3_N_4_ exhibits excellent photodegradation stability. Additionally, the comparison of RhB before and after the photocatalytic reaction, shown in Figure 6c, clearly demonstrates the exceptional degradation efficiency.

In this study, the photocatalytic performance of various catalysts for RhB removal was systematically evaluated. As shown in Table 1, the synthesized 1:2 Cl-C_3_N_4_ photocatalyst demonstrated a significant advantage, achieving 99.93% RhB degradation in just 80 min under simulated solar irradiation. Compared to the other catalysts in the study, it exhibited superior degradation efficiency and reaction kinetics.

### 2.4. Degradation Mechanism

#### 2.4.1. Radical Trapping and EPR Tests

**Figure 7:**
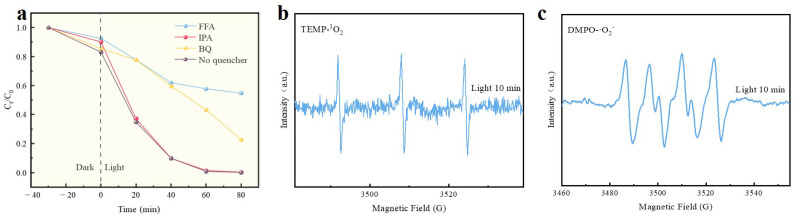
(**a**) Effect of different bursting agents on the degradation of RhB by 1:2 Cl-C_3_N_4_; (**b**) EPR of DMPO– ·O_2_^−^ (**c**) EPR of TEMP– ^1^O_2_.

Figure 7a illustrates the photodegradation of 1:2 Cl-C_3_N_4_, assessed through trapping experiments with different agents (IPA, BQ, FFA) under optimal conditions to further investigate the types of reactive oxygen species (ROS) involved in the photodegradation process. IPA serves as a trap for hydroxyl radicals (·OH), FFA acts as a trap for singlet oxygen (^1^O_2_), and BQ is used to capture superoxide radicals (·O_2_^−^) [59]. The results show that the addition of IPA led to a degradation rate of 99.62%, with minimal impact on the degradation rate, effectively excluding the involvement of ·OH. In contrast, the degradation rate of RhB decreased from 99.93% to 77.25% and 45.21% following the addition of BQ and FFA, respectively. These findings suggest that ·O_2_^−^ and ^1^O_2_ are the primary active species in the photocatalytic degradation of RhB. Under acidic conditions, dissolved oxygen is more readily able to accept electrons from the surface of the material, generating superoxide radicals, which are partially converted into singlet oxygen [60,61], thereby enhancing the degradation of RhB. EPR analysis further corroborated the active species involved in the RhB degradation process within the 1:2 Cl-C_3_N_4_ system. To capture these species, 5,5-Dimethyl-1-pyrroline N-oxide (DMPO) and 2,2,6,6-tetramethyl-4-piperidone (TEMP) were employed as trapping agents [62]. As depicted in Figure 7b,c, two distinct EPR signals were detected following 10 min of visible light irradiation. The intensity ratios of these signals, 1:1:1 and 1:2:2:1, are attributed to the characteristic peaks corresponding to TEMP– ^1^O_2_ and DMPO– ·OH, respectively.

#### 2.4.2. Photodegradation Mechanism of 1:2 Cl-C_3_N_4_ Toward RhB

The analysis, performed through free radical trapping experiments, proposes a potential reaction mechanism for the degradation of RhB by 1:2 Cl-C_3_N_4_. Upon visible light irradiation, 1:2 Cl-C_3_N_4_ absorbs photons, exciting electrons from the valence band (VB) to the conduction band (CB), thereby generating electron-hole pairs. The electron (e^−^) in the conduction band subsequently reacts with adsorbed oxygen (O_2_) to form superoxide radicals (·O_2_^−^). Simultaneously, the hole (h^+^) in the valence band oxidizes adsorbed water (H_2_O) or hydroxyl groups (OH^−^) on the surface, leading to the formation of hydroxyl radicals (·OH). The superoxide radical (·O_2_^−^) further interacts with either the hydroxyl radical (·OH) or the hole (h^+^), ultimately resulting in the generation of singlet oxygen (^1^O_2_) in its linear state [63,64]. As the primary reactive species, both the superoxide radical (·O_2_^−^) and singlet oxygen (^1^O_2_) effectively attack the RhB molecule, facilitating its degradation into carbon dioxide (CO_2_), water (H_2_O), and smaller molecules. The potential reactions involved in this mechanism are outlined in the following equations:

Cl-C_3_N_4_ + hv → h^+^_VB_ + e^−^_CB_ (1)

e^−^_CB_ + O_2_ → ·O_2_^−^ (2)

h^+^_VB_ + H_2_O → ·OH + H^+^ (3)

h^+^_VB_ + OH^−^ → ·OH (4)

·O_2_^−^ + ·OH/h^+^_VB_ → ^1^O_2_ (5)

·O_2_^−^/^1^O_2_ + RhB → CO_2_ + H_2_O + small molecules (6)

## 3. Experimental Section

### 3.1. Reagents and Instruments

#### 3.1.1. Experimental Reagents

Melamine, ammonium chloride, RhB, ethanol, sodium hydroxide, concentrated hydrochloric acid, furfuryl alcohol (FFA), isopropyl alcohol (IPA), p-benzoquinone (BQ), 5,5-Dimethyl-1-pyrroline N-oxide (DMPO), 2,2,6,6-tetramethyl-4-piperidone (TEMP), and deionized water.

#### 3.1.2. Experimental Equipment

Electronic Analytical Balance (Sartorius, Göttingen, Germany), Muffle Furnace (Hengzhida, Guangzhou, China), PH Meter (Lichen, Shaoxing, China), Visible Light Photocatalyzer (CEL-LAB500E4, CEALIGHT, Beijing, China), Ultrasonic Cleaning Instrument (CR-060S, Chunrain, Shenzhen, China), Electrothermal Drum Dryer (DHG-9123A, Jinghong, Shanghai, China), UV-5500PC UV Photometer, Steady-state/transient fluorescence spectrometer (Edinburgh FLS1000, Edinburgh, UK), X-ray diffractometer (Rigaku Miniflex 600, Akishima, Japan), Scanning Electron Microscope SEM (ZEISS Sigma 300, Jena, Germany), X-ray photoelectron spectrometer (Thermo Scientific K-Alpha, Waltham, MA, USA), UV-Vis Diffuse Reflectance Spectrometer (Shimadzu UV-2600, Kyoto, Japan), Automatic Specific Surface and Porosity Analyzer (Micromeritics, Norcross, GA, USA), Electrochemical workstation (Princeton, AMETEK Scientific Instruments, Oak Ridge, TN, USA), Magnetic and Resonance Spectrometer EPR (Bruker EMXplus-6/1, Karlsruhe, Germany), TOC analyzer (TOC-V CPH, Shimadzu, Japan), and photocatalytic reaction chamber (CEL-LB70, China Education Au-light Co., Ltd., Beijing, China).

### 3.2. Experimental Process

#### 3.2.1. Preparation of Photocatalysts

##### Preparation of g-C_3_N_4_

Using the thermal polymerization method, a certain amount of melamine was placed into a crucible, which was then wrapped in tinfoil to prevent exposure to air. The crucible was subsequently placed in a muffle furnace for calcination. The temperature was increased at a rate of 5 °C/min, reaching 550 °C. At this temperature, the material was held for 3 h, followed by natural cooling to room temperature. The resulting yellowish product was then ground into a powder, yielding g-C_3_N_4_.

##### Preparation of Cl-C_3_N_4_

Six grams of melamine were weighed and combined with specific amounts of ammonium chloride (1.2 g, 3 g, 6 g, and 12 g). The mixture was thoroughly ground in a mortar to ensure homogeneity, then transferred to a crucible, sealed with aluminum foil, and placed in a muffle furnace. The temperature was increased at a rate of 5 °C/min until it reached 550 °C, where it was maintained for 3 h. After cooling to room temperature, the resulting solid was ground into a fine powder. The modified catalysts were prepared at mass ratios of ammonium chloride to melamine of 1:5, 1:2, 1:1, and 2:1, respectively.

#### 3.2.2. Photocatalytic Experiment

RhB solution was selected as a model organic dye pollutant to evaluate the photocatalytic degradation performance of the samples. A precisely measured amount of Cl-doped graphitic carbon nitride (Cl-C_3_N_4_) catalyst, within the concentration range of 10–100 mg/L, was introduced into 100 mL of RhB solutions with varying pollutant concentrations (20–50 mg/L). Prior to initiating the photocatalytic reaction, the suspension was placed in a dark environment and stirred magnetically for 30 min to achieve adsorption–desorption equilibrium between the catalyst surface and the RhB molecules. The photocatalytic reaction was then carried out under continuous irradiation for 80 min using a 300 W xenon lamp (wavelength range: 380–830 nm), with magnetic stirring maintained throughout to ensure uniform dispersion of the catalyst. At 20-min intervals, 3 mL of the reaction solution was withdrawn, and the absorbance was measured at the maximum absorption wavelength of 554 nm after filtration through a 0.45 μm microporous filter membrane. The degradation efficiency (η) of RhB was calculated using the following Equation (7):

η = (C_0_ − C)/C_0_ × 100% (7)

where C_0_ and C are the initial and reaction t-time mass concentrations of RhB in mg/L, respectively; η is the clearance of RhB in %.

#### 3.2.3. Reactive Group Capture Probe

IPA, FFA, and BQ were used as the masking agents for hydroxyl radicals (·OH), singlet oxygen (^1^O_2_), and superoxide radicals (·O_2_^−^), and the reactive group capture experiments were carried out by adding 1 mmol/L of the masking agent to investigate the degree of activity of the reactive groups and their roles.

### 3.3. Testing and Characterization

Sample morphology was characterized using a (ZEISS Sigma 300) scanning electron microscope (SEM). X-ray diffractograms (XRD) were recorded by an X-ray diffractometer (Rigaku Miniflex 600, Japan) to analyze the crystal structure and phase composition of the materials, using Cu Kα radiation (λ = 0.15406 nm) at a scanning speed of 5°/min and a scanning angle ranging from 10° to 80°. Photoluminescence (PL) spectra of the samples were measured using a steady-state/transient fluorescence spectrometer (Edinburgh FLS1000, UK) with an excitation wavelength of 325 nm, which can reveal the carrier complexation mechanism in the material. The chemical composition and chemical states of the X-ray photoelectron spectra (XPS) were characterized using an X-ray photoelectron spectrometer (Thermo Scientific K-Alpha, USA), and the measured binding energies of the elements were corrected using the C1s carbon peak at 284.8 eV as a reference. The specific surface area, pore size distribution, and pore structure of the materials were determined by BET analysis using an automatic surface area and porosity analyzer (Micromeritics, USA) with nitrogen adsorption at 77 K. Ultraviolet-visible diffuse reflectance spectroscopy (UV-vis DRS) was recorded using a Shimadzu UV 2600 spectrophotometer (Shimadzu Co., Kyoto, Japan) in the range of 200 nm to 800 nm to analyze the light absorption properties of the materials. The electrochemical workstation, CHI 660D (Princeton, Bee Cave, TX, USA), was employed in a three-electrode system, where the working electrode was prepared by the drop-coating method, with Ag/AgCl as the reference electrode and Pt as the counter electrode. The electrolyte used was 0.1 M Na_2_SO_4_. The working electrode was prepared by weighing 2.5 mg of the catalyst and 50 µL of Nafion solution, followed by dispersion through ultrasonication with the addition of 0.45 mL of a water and ethanol mixture (1:9 volume ratio). A 20 µL drop of the dispersion was pipetted onto conductive glass (1 × 1 cm^2^) and dried in a natural environment. Transient photocurrent tests were conducted using a Princeton Versa STAT 4 electrochemical workstation (AMETEK Scientific Instruments). By monitoring the current response of the material under light illumination, its photoelectric conversion performance was evaluated, and the separation, migration, and recombination behaviors of photogenerated carriers were analyzed. Electron paramagnetic resonance (EPR) was measured using a magnetic and resonance spectrometer (Bruker EMXplus-6/1, Germany) to investigate the free radicals generated during photocatalysis. The total organic carbon (TOC) content was measured using a TOC analyzer (TOC-V CPH, Shimadzu, Japan).

## 4. Conclusions

The Cl-C_3_N_4_ composite was synthesized via a thermal polymerization method, using melamine as the precursor and ammonium chloride as the dopant. The photocatalytic performance of Cl-C_3_N_4_, with varying mass ratios, was investigated for the degradation of RhB, a model organic pollutant. The results demonstrated that the Cl-C_3_N_4_ composite with a mass ratio of 1:2 exhibited the highest photocatalytic performance, achieving a degradation efficiency of up to 99.93%. Even after four repeated cycles, the degradation rate remained above 88%. In the photocatalytic degradation process of RhB, the reactive species ·O_2_^−^ and ^1^O_2_ played the predominant roles, with the following order of activity: ^1^O_2_ > ·O_2_^−^ > ·OH. This provides valuable theoretical insights into the reaction mechanism of Cl-C_3_N_4_ photocatalysts. The composite material enhanced the separation and migration of charge carriers, thereby overcoming the limitations of the single catalyst. The prepared 1:2 Cl-C_3_N_4_ photocatalyst holds great potential for application in organic wastewater treatment.

Although chlorine doping significantly improved the photocatalytic performance of g-C_3_N_4_, its long-term stability and durability in practical environmental conditions require further investigation. Future research may explore the co-doping of chlorine with other non-metallic elements (such as sulfur, phosphorus, etc.) to further optimize the material’s photocatalytic performance. Through the synergistic effect of multiple elements, it may be possible to achieve a more efficient charge carrier separation and a broader light response range. The impact of non-metallic element doping on the electronic structure, band alignment, and the formation pathways of reactive species in g-C_3_N_4_ can also be further explored using computational methods such as density functional theory (DFT), which would provide valuable theoretical guidance for material design.

## Figures and Tables

**Table 1:** Comparison of the removal effect of RhB by different photocatalysts.

Photocatalyst	Dosage Amount (mg)	C_RhB_ (mg·L^−1^)	Light	Degradation Rate (%)	Free Radicals	Reference
BiOBr/ZIF-67	20	20	Visible light	95.2 (120 min)	·O_2_^−^, h^+^	[54]
COFs-Ph/CdS	20	20	Visible light	83 (100 min)	·O_2_^−^, ·OH	[55]
MoO_3_	30	10	UV-vis	96.2 (70 min)	·O_2_^−^, ·OH	[56]
Bi_2_O_3_/BiOCl	50	10	Visible light	96.8 (120 min)	·O_2_^−^, ·OH	[57]
3%Au/Bi_2_WO_6_	200	4.79	Visible light	96.2 (240 min)	h^+^, ·OH	[58]
CI/g-C_3_N_4_	25	20	Visible light	99.93 (80 min)	·O_2_^−^, ^1^O_2_	This work

## Data Availability

Data are contained within the article.
